# Effect of preoperative versus postoperative use of transversus abdominis plane block with plain 0.25 % bupivacaine on postoperative opioid use: a retrospective study

**DOI:** 10.1186/s12871-021-01333-6

**Published:** 2021-04-12

**Authors:** Richard Kalu, Peter Boateng, Lauren Carrier, Jaime Garzon, Amy Tang, Craig Reickert, Amalia Stefanou

**Affiliations:** 1 Department of Surgery, Henry Ford Hospital, 2799 W. Grand Blvd, MI 48202 Detroit, USA; 2 Department of Anesthesiology, Henry Ford Hospital, 2799 W. Grand Blvd, 48202 Detroit, MI USA; 3 Department of Public Health Sciences, Henry Ford Health System, One Ford Place, 48202 Detroit, MI USA; 4 Division of Colon and Rectal Surgery, Henry Ford Hospital, 2799 West Grand Blvd Detroit, 48202 Detroit, MI USA

**Keywords:** Transversus abdominis plane block, Preemptive analgesia, TAP block

## Abstract

**Background:**

Enhanced recovery protocols optimize pain control via multimodal approaches that include transversus abdominis plane (TAP) block. The aim of this study was to evaluate the effect of preoperative vs. postoperative plain 0.25 % bupivacaine TAP block on postoperative opioid use after colorectal surgery.

**Methods:**

A retrospective cohort study comparing postoperative opioid use in patients who received preoperative (*n* = 240) vs. postoperative (*n* = 22) plain 0.25 % bupivacaine TAP blocks. The study was conducted in a single tertiary care institution and included patients who underwent colorectal resections between August 2018 and January 2020. The primary outcome of the study was postoperative opioid use. Secondary outcomes included operative details, length of stay, reoperation, and readmission rates.

**Results:**

Patients who received postoperative plain 0.25 % bupivacaine TAP blocks were less likely to require postoperative patient-controlled analgesia (PCA) (59.1 % vs. 83.3 %; *p* = 0.012) and opioid medications on discharge (6.4 % vs. 16.9 %; *p* = 0.004) relative to patients who received preoperative TAP. When needed, a significantly smaller amount of opioid was prescribed to the postoperative group (84.5 vs. 32.0 mg, *p* = 0.047). No significant differences were noted in the duration of postoperative PCA use, amount of oral opioid use, and length of stay.

**Conclusions:**

Plain 0.25 % bupivacaine TAP block administered postoperatively was associated with significantly lower need for postoperative PCA and discharge opioid medications. The overall hospital length of stay was not affected by the timing of TAP block. Because of the limited sample size in this study, conclusions cannot be generalized, and more research will be required.

## Background

Enhanced recovery protocols (ERP) after surgery have the aim of reducing morbidity and the surgical stress response while advancing early return of patients to their baseline functioning [1]. Components of ERP include optimal pain control and surgical stress reduction with regional anesthesia, early mobilization, and early enteral nutrition [2, 3]. Multiple studies including randomized controlled trials have shown a reduction in hospital length of stay, duration of postoperative ileus, reduced morbidity, and an earlier return of normal function after ERP implementation [1, 4–6].

Optimal pain management is an integral part of an ERP, particularly after colon surgery. Poor pain control may lead to longer length of stay, cost, and patient dissatisfaction [6]. Many ERPs use a multimodal approach to achieve an optimal pain control, employing neuraxial and regional anesthesia techniques and lower utilization of opioids as the primary analgesic [7–9]. Transversus abdominis plane (TAP) block is an example of a regional anesthetic technique that has been used extensively in abdominal surgery [10, 11]. The TAP block involves injecting plain 0.25 % bupivacaine into the fascial plane between the internal oblique and transversus abdominis muscles. The duration of action for plain bupivacaine ranges from 2 to 10 h with peak effect noted around 30 to 45 min {Beiranvand, 2018 #348} [12].

A TAP block may be administered at any time during the immediate perioperative period. However, whether plain bupivacaine TAP block is effective in colorectal surgery remains to be elucidated. There have been no studies assessing the timing of TAP block administration for optimal postoperative pain control. In the present study, we assessed the effect of preoperative vs. postoperative administration of TAP block using plain 0.25 % bupivacaine on postoperative opioid use in patients undergoing colorectal surgery. Furthermore, we assessed for any effects on length of stay, rates of reoperation, and readmission.

## Methods

### Patient selection

All patients who received TAP blocks during a transabdominal colorectal resection between August 2018 and January 2020 were identified through hospital chart review. The TAP procedure was performed by the regional anesthesiologist on duty that day. The majority of TAP blocks are performed preoperatively in our institution. At times, the TAP block was done at the conclusion of the operation often due to timing issues. All included patients underwent colorectal resection done by colon and rectal surgeons at our tertiary care hospital. We abstracted patient demographics, medical comorbidities, preoperative diagnosis, past medical and surgical history, procedure-related details, and postoperative opioid use after the index operation until discharge. The study was approved by the Henry Ford Health System Institutional Review Board. This manuscript was conducted in accordance with the ethical standards laid down in the amended 1964 Declaration of Helsinki.

Patients were divided in 2 groups based on timing of TAP block procedure: before (preoperative) or immediately after (postoperative) their surgical procedure.

### TAP block technique

Under ultrasound guidance, TAP blocks were performed by, or under supervision of, the attending anesthesiologist who specializes in acute pain management and regional anesthesia. The blocks were performed either 1 h preoperatively or at the conclusion of the surgery procedure within 30 min of reaching the post-anesthesia care unit. The TAP block consisted of bilateral injection of 20 mL of 0.25 % plain bupivacaine in the fascial plane between the internal oblique and transversus abdominis muscles in the midaxillary line. None of the patients in the 2 groups received additional regional anesthesia, including epidural or spinal anesthesia.

### Additional multimodal adjuncts

Our postoperative pain management protocol consisted of scheduled acetaminophen, muscle relaxant (methocarbamol or cyclobenzaprine), gabapentin, and ketorolac in the absence of any contraindication. Patient-controlled analgesia (PCAs) were initiated for patients who had inadequate pain control, had pain-related hypertension or tachycardia, or were unable to ambulate or participate in pulmonary toilet. All patients were routinely assessed for their pain levels. However, the use of numerical pain scoring was not consistent amongst the patients and hence, was not included in the study. While there may be minor differences in pain management based on specific surgeon-preference, most of the patients were treated using this protocol. Finally, the decision to prescribe discharge opioid was based on several factors such as surgeon’s discretion, the level patient’s pain control, and history of chronic opioid use.

### Outcomes

The primary outcome of the study was postoperative opioid use. The incidence, type, and total amount of opioid in milligram morphine equivalent (MME) and non-opioid analgesic medications administered after the index procedure were recorded. Secondary outcomes included procedure-related operative details, length of hospital stay, rates of readmission, and reoperation.

### Statistical analysis

Statistical analyses were conducted to compare the baseline characteristics of the patients who received preoperative TAP blocks with those who received postoperative TAP blocks. Continuous variables were described using the mean and standard deviation (SD), and categorical variables were described with the frequency and percentage. Analysis of variance or Kruskal–Wallis tests were used for continuous variables and chi-square tests or Fisher’s exact tests were used for categorical variables as appropriate. *P* < 0.05 was considered statistically significant. All analyses were done in SAS 9.4 (SAS Institute, Cary, NC).

## Results

### Descriptive analysis

A total of 262 patients were identified through chart review. A total of 240 patients received preoperative TAP blocks and 22 received postoperative TAP blocks. The mean (SD) patient age was 57.8 years (16 years), 45 % were men, and the mean (SD) body mass index was 28.4 kg/m^2^ (7.32 kg/m^2^). There were no significant differences in the 2 groups with regard to age, sex, body mass index, American Society of Anesthesiology classification, history of cancer or inflammatory bowel disease, and opioid use at the time of the index procedure (Table 1). The 2 groups were similar in terms of comorbidities, including history of hypertension, diabetes mellitus, hyperlipidemia, congestive heart failure, chronic pulmonary obstructive disease, smoking, and alcohol use. The surgical indications and surgical approaches were similar between the 2 groups (Table 1).

**Table 1:** Patient characteristics based on timing of plain transversus abdominis plane (TAP) block

Variables	Overall (*N* = 262)	Preoperative TAP (*n* = 240)	Postoperative TAP (*n* = 22)	*P*-value
Age, years, mean (SD)	57.76 (16.10)	57.83 (15.87)	56.95 (18.90)	0.807
Male, no. (%)	118 (45.0)	107 (44.6)	11 (50.0)	0.791
Body mass index, kg/m^2^, mean (SD)	28.38 (7.32)	28.51 (7.48)	27.03 (5.27)	0.366
Diabetes mellitus, no. (%)	67 (25.6)	64 (26.7)	3 (13.6)	0.278
Hyperlipidemia, no. (%)	156 (59.5)	144 (60.0)	12 (54.5)	0.786
Cigarette smoking, no. (%)	85 (32.4)	80 (33.3)	5 (22.7)	0.436
History of COPD, no. (%)	21 (8.0)	19 (7.9)	2 (9.1)	0.692
Alcohol use, no. (%)	126 (48.1)	113 (47.1)	13 (59.1)	0.392
Hypertension, no. (%)	132 (50.4)	121 (50.4)	11 (50.0)	0.99
History of CHF, no. (%)	22 (8.4)	21 (8.8)	1 (4.5)	0.705
Current opioid use, no. (%)	43 (16.5)	41 (17.2)	2 (9.1)	0.499
Steroid use, no. (%)	37 (14.1)	31 (12.9)	6 (27.3)	0.126
Previous abdominal surgery, no. (%)	170 (64.9)	157 (65.4)	13 (59.1)	0.718
Blood thinner use, no. (%)				
Aspirin	52 (19.8)	47 (19.6)	5 (22.7)	0.941
Warfarin	7 (2.7)	6 (2.5)	1 (4.5)	0.463
Plavix	11 (4.2)	11 (4.6)	0 (0.0)	0.607
Others	25 (9.5)	22 (9.2)	3 (13.6)	0.451
ASA class, no. (%)				0.241
ASA class 1	1 (0.4)	1 (0.4)	0 (0.0)	
ASA class 2	85 (32.4)	78 (32.5)	7 (31.8)	
ASA class 3	169 (64.5)	156 (65.0)	13 (59.1)	
ASA class 4	7 (2.7)	5 (2.1)	2 (9.1)	
ASA > 2	176 (67.2)	161 (67.1)	15 (68.2)	0.99
Surgical indication, no. (%)				
Malignancy	129 (49.2)	119 (49.6)	10 (45.5)	0.882
IBD	52 (19.8)	46 (19.2)	6 (27.3)	0.401
Benign disease	128 (48.9)	116 (48.3)	12 (54.5)	0.738

*ASA* American Society of Anesthesiology, *COPD* chronic obstructive pulmonary disease, *CHF *congestive heart failure, *IBD* inflammatory bowel disease, *SD* standard deviation

### Analgesic Requirements

Table 2 shows the postoperative analgesics used by the two groups. The patients who received plain bupivacaine TAP blocks postoperatively experienced a statistically significant reduction in the overall use of PCA compared with those who received preoperative TAP blocks (59.1 % vs. 83.3 %; *p* = 0.012). However, when given a PCA, the postoperative TAP group used a significantly higher amount of morphine compared to their counterparts (30.77 MME vs. 27.09 MME; *p* = 0.019). The postoperative TAP group was less likely to be prescribed opioid medication at the time of discharge (6.4 % vs. 16.9 %; *p* = 0.004). For patients who received prescription opioid at the time of discharge, the patients who had postoperative TAP received a significantly smaller amount of opioid (128.09 MME vs. 73.64 MME; *p* = 0.047). There were no differences between the groups with regard to duration of PCA or intravenous and oral opioid use.

**Table 2:** Postoperative analgesics use based on when the plain TAP block was administered

Variables	Overall (*N* = 262)	Preoperative TAP (*n* = 240)	Postoperative TAP (*n* = 22)	*P*-value
PCA use, no. (%)	213 (81.3)	200 (83.3)	13 (59.1)	0.012
PCA type				0.003
Morphine	172 (65.6)	165 (68.8)	7 (31.8)	
Hydromorphone	31 (11.8)	26 (10.8)	5 (22.7)	
Morphine + hydromorphone	10 (3.8)	9 (3.8)	1 (4.5)	
PCA amount, MME, mean (SD)				
Morphine	27.40 (57.37)	27.09 (51.87)	30.77 (101.32)	0.019
PCA duration, hours, mean (SD)	33.97 (64.30)	34.66 (66.22)	26.36 (37.48)	0.263
IV opioid use, no. (%)	54 (20.6)	46 (19.2)	8 (36.4)	0.102
IV opioid amount, mg, mean (SD)	1.80 (10.39)	1.38 (9.07)	6.42 (19.50)	0.051
Oral opioid amount, MME, mean (SD)	93.94 (153.61)	87.71 (117.82)	161.93 (360.53)	0.743
Discharge opioid, MME, mean (SD)	123.52 (123.01)	128.09 (126.78)	73.64 (48.03)	0.005
Prescription muscle relaxants on discharge, no. (%)	108 (41.4)	97 (40.6)	11 (50.0)	0.528
Opioid use on first postoperative follow-up, no. (%)	19 (7.3)	18 (7.5)	1 (4.5)	0.99
Opioid refill request at first postoperative follow up, no. (%)	3 (1.1)	2 (0.8)	1 (4.5)	0.605

*IV* intravenous, *PCA* patient-controlled analgesia, *SD* standard deviation, *TAP* transversus abdominis plane, *MME *morphine milligram equivalent

### Procedure-related details, length of stay, reoperation, and readmission

Table 3 presents the procedure-related details, length of stay, and reoperation and readmission rates for the 2 groups. Surgical approach did not differ based on timing of the regional anesthesia. There was no statistically significant difference in procedure length, estimated blood loss, length of hospital stays, reoperation, or readmission rates between the 2 groups.

**Table 3:** Procedure-related details, length of stay, reoperation, and readmission

Variables	Overall (*N* = 262)	Preoperative TAP (*n* = 240)	Postoperative TAP (*n* = 22)	*P*-value
Surgical procedure, no. (%)				0.554
Surg_type1^a^	195 (74.4)	178 (74.2)	17 (77.3)	
Surg_type2^b^	38 (14.5)	36 (15.0)	2 (9.1)	
Surg_type3^c^	11 (4.2)	9 (3.8)	2 (9.1)	
Surg_type4^d^	18 (6.9)	17 (7.1)	1 (4.5)	
Surgical approach, no. (%)				0.439
Open	69 (26.3)	62 (25.8)	7 (31.8)	
Laparoscopic	79 (30.2)	75 (31.2)	4 (18.2)	
Robotic	114 (43.5)	103 (42.9)	11 (50.0)	
Procedure details				
Emergent procedure, no. (%)	2 (0.8)	1 (0.4)	1 (4.5)	0.161
Procedure length, minutes (SD)	208.38 (96.27)	207.79 (97.16)	214.82 (87.76)	0.655
EBL in mL, mean (SD)	100.05 (185.54)	100.43 (190.96)	95.91 (113.14)	0.855
LOS in days, mean (SD)	4.92 (5.95)	4.82 (5.62)	6.00 (8.87)	0.836
Readmission rate, no. (%)	29 (11.2)	28 (11.8)	1 (4.5)	0.485
Reoperation rate, no. (%)	5 (1.9)	4 (1.7)	1 (4.5)	0.901

*EBL* estimated blood loss, *LOS* length of day, *SD* standard deviation, *TAP* transversus abdominis plane

^a^surg_type1 includes hemicolectomy, sigmoidectomy, low anterior resection, total abdominal colectomy and abdominoperineal resection

^b^surg_type2 includes ostomy reversal

^c^surg_type3 includes ostomy creation

^d^surg_type4 includes appendectomy, exploratory laparotomy, lysis of adhesion, and rectopexy

## Discussion

Postoperative pain management is an integral part of achieving the goals of ERPs after colorectal surgery. TAP blocks are an attractive approach for minimizing the use of opioids, especially given their low risk of adverse effects. Although usually done preoperatively, our study showed that postoperative TAP block with plain bupivacaine appeared to be at least as efficacious as preoperative TAP block in reducing postoperative intravenous opioid use, both PCA and administered intravenous injections. Furthermore, postoperative plain TAP block was associated with a reduced total amount of prescription opioids needed to the time of discharge from the hospital. Other variables such as length of stay, estimated operative blood loss, procedure length, reoperation and readmission rates did not differ between preoperative or postoperative administration of the TAP.

The effectiveness and feasibility of TAP blocks in colorectal surgery has been shown in multiple studies [7, 13–17]. In a randomized, placebo-controlled clinical trial, Tikuisis et al. showed that patients who received ropivacaine TAP blocks had significantly lower pain scores at 2, 4, and 12 h at rest, and at 2- and 4-hours during movement. The TAP group also used significantly less fentanyl and ketorolac following a hand-assisted laparoscopic left hemicolectomy for colon cancer compared to those who received placebo [13]. Pirrera et al. compared the use of preoperative ropivacaine TAP block vs. thoracic epidural analgesia in patients before elective laparoscopic colon resection. Both patient groups were a part of a standard enhanced recovery after surgery pathway. Albeit a case-control study, pain control was comparable between the 2 groups. Additionally, the TAP group had significantly lower rates of postoperative nausea, vomiting, ileus, and paresthesia. There was no significant difference in hospital length of stay or 30-day readmission rate. In a prospective, randomized, double-blind study, Keller et al. assessed the effect of TAP blocks on postoperative pain in patients following laparoscopic colorectal resections. Compared to their counterparts, the TAP group had significantly lower pain scores and used fewer opioids. However, there was no difference in hospital length of stay and readmission rate between the groups [17]. These research study findings are consistent with our current results.

Considering the short duration of action of plain bupivacaine, the timing of its administration for TAP blocks can be planned to provide optimal analgesia. Preoperative administration is easily performed in the preoperative area and does not affect surgical planning such as ostomy procedures. It is also in line with preemptive analgesia. Oliveira et al., through a meta-analysis that included a variety of abdominopelvic surgeries, reported a greater postoperative pain control, reduced pain at rest, and decreased opioid used with preoperative TAP compared to placebo or no treatment [18]. The majority of the TAP blocks performed in our study were performed preoperatively, but we found more benefit with postoperative TAP blocks. While the numbers are small in the postoperative TAP group, the postoperative TAP group did not show inferior pain control compared to preoperative administrated block. In fact, our results showed that plain bupivacaine TAP administered postoperatively led to a reduced postoperative intravenous opioid use and lesser amount of discharge prescription opioid.

The study was limited by its retrospective design, the small number of patients who received postoperative plain bupivacaine TAP block procedures, and by having been performed in a single center. Although the TAP blocks were performed by, or under supervision of, the attending anesthesiologists who specialize in acute pain management and regional anesthesia, we acknowledge the possibility that performer-related differences could lead to differences in pain relief. However, the regional block was administered by well-trained and experienced anesthesiologists who routinely perform the procedure, hence minimizing the effect of performer-related differences. Finally, our study did not directly measure the patient pain scores. Instead, we used the amount of postoperative opioid used as a surrogate for the adequacy of pain control. Per our ERP, additional analgesia is prescribed on an as-needed basis and coincides with the patient’s assessment of pain on a numeric scale.

## Conclusions

TAP blocks provide an effective and feasible means of optimizing pain control in the era of enhanced recovery after surgery. When administered postoperatively, plain bupivacaine TAP may be as effective as preoperative TAP blocks, offering the same analgesic effect and minimizing intravenous opioid use.

## Data Availability

The datasets used and/or analyzed during the current study are not publicly available due to patient privacy and institutional policy but are available from the corresponding author on reasonable request.

## Abbreviations

ERP: Enhanced recovery protocols
PCA: Patient-controlled analgesia
TAP: Transversus abdominis plane
